# Michigan’s New Dental Law

**Published:** 1907-07-15

**Authors:** 


### MICHIGAN'S NEW DENTAL LAW.

Section 1. It shall be unlawful for any person not a registered dentist within the meaning of this act, to practice dentistry or dental surgery in any of its departments, as principal or agent, in the State of Michigan, except as hereinafter provided.

Sec. 2. From and after the passage of this act, the State Board of Dental Examiners herein provided for, shall consist of five members, to be appointed by the Governor. The members of the board created by act number one hundred and forty of the Public Acts of eighteen hundred and eighty-three, entitled "An act to regulate the practice of dentistry in the State of Michigan," holding office at the time of passage of this act, shall be continued in office as members of the State Board of Dental Examiners until expiration of the term for which they are appointed. The additional members of said board shall be appointed for such periods that the term of office of one member of said board shall expire annually, and annually on January 1st thereafter, the Governor shall appoint a member of said board whose term of office shall be for a period of five years. The several members of said board shal hold office until their respective successors are appointed and qualified, and if any vacancy occurs in said board, another shall be appointed as aforesaid to fill the unexpired term thereof. The members of said board shall be electors and reputable dentists, who have resided in this State for at least five years and have had at least five years' experience in their profession. Said board shall have full power to make by-laws and necessary regulations for the proper fulfillment of their duties under this act. It shall choose one of its members president and one secretary-treasurer, and shall hold two regular meetings each year, at such dates and places as may be deemed best. Special meetings may also be held. A majority of the board shall constitute a quorum for the transaction of business. Said board shall keep a full record of its proceedings and a full registry of all persons licensed and certified as dentists by said board, which shall be public records, and at all times open to inspection as such. A transcript of any of the entries in such record, certified by the secretary-treasurer under the seal of said board, shall, at all times and places, be competent evidence of the facts therein stated. The members of said board shall have the power to administer oaths and hear testimony in all matters relatng to the duties imposed upon it bylaw. Said board shall make an annual report of its proceedings to the Governor on or before the thirty-first day of December in each year.

Sec. 3. All persons who desire to begin the practice of dentistry in this State after the passage of this act, and who shall have a license from the dental board of another state, or who shall have received a diploma from the faculty of some reputable dental college duly organized under the laws of this or any other State of the United States, shall have the right to apply to the dental board of this State for examination as to their proficiency; and all successluf applicants shall be licensed and registered by said dental board. Said dental board shall be authorized to ascertain and determine what shall constitute a dental college or institution in good standing and repute; but no such dental college or dental institution shall be considered reputable unless the same shall possess the following qualifications:

First, It shall be chartered under the laws of the State in which it is located and operated, and shall be authorized by its charter to confer the degrees of Doctor of Dental Surgery or Doctor of Dental Medicine.

Second, It shall deliver, annually, a full course of lectures and instructions by a competent faculty and corps of instructors in the following subjects: Anatomy, chemistry, physiology, histology, materia medica, therapeutics, dental metallurgy, pathology, bacteriology, operative dentistry, prosthetic dentistry, crown and bridge work and oral surgery and hygiene, said course of instruction to consist of not less than three terms in separate academic years and of not less than thirty-two weeks of six days for each session, and shall require its matriculates to have a general education equivalent to that required for graduation from a high school of recognized standing;

Third, The apparatus and equipment of each said dental college or institution shall be ample and sufficient for the ready and full teaching of the above named subjects, and every such college shall allow said State Board of Dental Examiners of this State the privilege of inspecting its work and equipment at any time.

Sec. 4. Said dental board shall have power, after due notice in writing for twenty days and upon a full hearing at a time and place fixed in said notice, to revoke and annul any original license or registration for fraud, deceit or misrepresentation in the practice of dentistry, or for gross violations of professional duties. Nor shall said dental board re-license any one whose license has been revoked for any of the above causes within one year after such revocation, and then only upon sufficient assurance and guarantees to said board of correct conduct for the future. The notice hereinbefore provided to be given may be served by registered letters mailed to the address of the dentist under investigation, and when the whereabouts of any such person is unknown, then said notice may be sent to the last known address of such person.

Sec. 5. All persons entitled to examination as provided in this act shall file application and license in writing, supported by affidavit, stating the facts which entitle him or her to such examination, and each applicant shall accompany his or her application with such license or diploma for verification as to its genuineness. All applicants for examination shall, at the time of making such application, pay to the secretary-treasurer of the dental board, a fee of twenty dollars, and each applicant shall present himself before the said dental board for examination at the first or second regular meeting after his application shall have been made, and in default thereof, said fee shall be forfeited to said dental board. The fee for any subsequent application for examination or re-examination shall be ten dollars. The examination may be written or oral or both, at the option of said board, and shall include the following subjects: Anatomy, chemistry, physiology, histology, bacteriology, operative dentistry, prosthetic dentistry, crown and bridge work, and oral surgery and hygiene. All persons of good moral character, who shall successfully pass such examination, shall be licensed and registered by said dental board and shall receive a certificate of such license and registration duly authenticated by the signature of the members of the board, and with the seal of said board attached; and in no case shall said examination fee be refunded, but said dental board may, for sufficient cause, remit said fee for subsequent re-examination.

Sec. 6. No person having received a certificate from the State Board of Dental Examiners in the manner hereinbefore provided, shall engage in the business of a dentist in any county of the State in which he shall locate, or into which he shall afterwards remove, until he shall have had such certificate recorded in the office of the county clerk of such county, and it is hereby made the duty of the county clerk to record such certificates in a book provided and kept for that purpose, and the clerk is authorized to charge a fee of fifty cents for recording such certificate, to be paid by the person offering such certificate, for record. The record of each certificate required by this act, or certified copy thereof, shall be evidence in all courts that the person holding it is a registered dentist within the meaning of this act. It is hereby made the duty of each county clerk in the State to furnish the State Board of Dental Examiners on the first day of July a list of all dentists registered in his county during the preceding year, this report to be made on tabulated blanks to be sent to said county clerk for the purpose. The register of the county clerk shall be open to public inspection during business hours. Any failure or neglect or refusal on the part of any person holding such certificate to register the same with the county clerk as above directed, for a period of six months, shall work a forfeiture of the certificate. In order that a complete register of all the dentists practicing in this State at the present time may be made, within sixty days after this act takes effect, it shall be the duty of every dentist at present practicing in the State, whether legally licensed at present or otherwise, to forward to the Board of Dental Examiners an affidavit setting forth the facts of his registration or the credentials upon which he may claim re-registration. This application shall be accompanied with a fee of three dollars, which shall be deposited in a special fund to be used only by the Board of Examinatrs for the enforcement of this act against unlicensed or unregistered practitioners. No licesne or certificate of registration when once forfeited, for any cause, shall be registered, except upon the payment to the said Board of Dental Examiners of the sum of twenty-five dollars as a penalty for such neglect, failure, refusal or mis conduct.

Sec. 7. All persons shall be said to be practicing dentistry within the meaning of this act, who shall use the words or letters "Dentist," "D. D. S.," or any other letters or title in connection with his name, which in any way represents him as engaged in the practice of dentistry, or who shall advertise or permit it to be done by sign, card, circular, handbill, newspaper or otherwise, that he can or will attempt to perform dental operations of any kind, treat disease or lesions of the human teeth or jaws, or replace lost teeth by artificial ones, or attempt to correct malposition thereof, or who shall for a fee, salary, or other reward, paid or to be paid, either to himself or to another person, perform dental operations of any kind, treat diseases or lesions of the human teeth or jaws, or replace lost teeth by artificial ones, or attempt to correct malposition thereof. But nothing contained in this act shall be taken as applying to the acts of legally qualified physicians in the extraction of teeth, in the performance of their duties as such, or to acts of bona fide students of dentistry done in the college building, in the pursuit of clinical advantages while in attendance upon a regular course of study in a reputable dental college. Any dentist, proprietor, partnership, association or corporation, owning, running, operating or controlling any room or rooms, office or dental parlors, where dental work is done, provided or contracted, who shall employ, keep or retain any unlicensed dentist or student as an operator shall be guilty of a misdemeanor and punished as in section nine.

Sec. 8. Out of the funds coming into the possession of said board as above specified, the members of said board may receive as compensation the sum of ten dollars for each day actually engaged in the duties of their office as such examiners and actual necessary expenses. Said expense shall be paid from the fees and assessments received by said board under the provisions of this act, and no part of the salary or expense of said board shall ever be paid out of the State treasury. All moneys received in excess of said per diem allowance and mileage as above provided for, shall be held by the secretary-treasurer of said board, as a special fund for other expenses of said board and for carrying out the provisions of this act. The secretary-treasurer of said board shall, from time to time, give such bond for the faithful discharge of his duties as the custodian of funds of said board as it may direct. Said board shall appropriate from any fund under its control, a sum of not to exceed five hundred dollars annually as compensation for the services of secretary-treasurer.

Sec. 9. Any person who shall practice, or attempt to practice, either as proprietor, employee or assistant, without having a license, or without having his license renewed as provided by section six of this act, or without keeping his license in open view in his operating room, shall be punished by a fine of not less than fifty dollars nor more than two hundred dollars, or by confinement in the county jail not less than twenty days, or by both such fine and imprisonment. It is hereby made the duty of the prosecuting attorney of each county in the State to prosecute every case to final judgment, whenever his attention shall be called to a violation of this act.

Sec. 10. An applicant shall be registered and given a certificate of registration if he or she present a certified copy of certificate of registration or license which has been issued to said applicant in any other state or foreign country where the requirements for registration shall be deemed by said board to be equivalent to those of this act: Provided, That such country or state shall accord a like privilege to holders of certificates from this board. The fee for registration of applicants of this class shall be ten dollars, to be paid at the time of application.

Sec. 11. Any person filing, or attempting to file, as his own, the diploma or license of another, or a forged affidavit of identification or qualification, shall be deemed guilty of a felony, and upon conviction thereof, shall be subject to such fine and imprisonment as is made and provided by the statutes of this State for the crime of forgery.

Sec. 12. Act No. 140 of the Public Acts of 1883, and all acts or parts of acts in any way contravening the provisions of this act are hereby repealed.

